# Characterization of Corticotropin-Releasing Hormone neurons in the Paraventricular Nucleus of the Hypothalamus of *Crh-IRES-Cre* Mutant Mice

**DOI:** 10.1371/journal.pone.0064943

**Published:** 2013-05-28

**Authors:** Jaclyn I. Wamsteeker Cusulin, Tamás Füzesi, Alan G. Watts, Jaideep S. Bains

**Affiliations:** 1 Hotchkiss Brain Institute and Department of Physiology and Pharmacology, University of Calgary, Calgary, Alberta, Canada; 2 Department of Biological Sciences, University of Southern California, Los Angeles, California, United States of America; University of Rouen, France, France

## Abstract

Corticotropin-releasing hormone (CRH)-containing neurons in the paraventricular nucleus of the hypothalamus (PVN) initiate and control neuroendocrine responses to psychogenic and physical stress. Investigations into the physiology of CRH neurons, however, have been hampered by the lack of tools for adequately targeting or visualizing this cell population. Here we characterize CRH neurons in the PVN of mice that express tdTomato fluorophore, generated by crosses of recently developed *Crh-IRES-Cre* driver and *Ai14* Cre-reporter mouse strains. tdTomato containing PVN neurons in *Crh-IRES-Cre;Ai14* mice are readily visualized without secondary-detection methods. These neurons are predominantly neuroendocrine and abundantly express CRH protein, but not other PVN phenotypic neuropeptides. After an acute stress, a large majority of tdTomato cells express neuronal activation marker c-Fos. Finally, tdTomato PVN neurons exhibit homogenous intrinsic biophysical and synaptic properties, and can be optogenetically manipulated by viral Cre-driven expression of channelrhodopsin. These observations highlight basic cell-type characteristics of CRH neurons in a mutant mouse, providing validation for its future use in probing neurophysiology of endocrine stress responses.

## Introduction

Real and perceived threats to survival engage an evolutionarily conserved endocrine stress response. Part of this response, mediated by a hypothalamic-pituitary-adrenal axis (HPA), culminates in the release of glucocorticoid hormones in to the blood, promoting critical body-wide adaptive changes [1]. At the head of the HPA axis is a cluster of parvocellular neuroendocrine cells (PNCs) in the paraventricular nucleus of the hypothalamus (PVN) [2]. Decades of careful anatomical and physiological study indicates that these neurons, through somatic production and release of secretogogues at fenestrated capillaries entering pituitary portal circulation, directly control pituitary corticotroph function, and downstream glucocorticoid secretion by the adrenal glands. The most widely studied of these is corticotropin releasing hormone (CRH) or factor (CRF), a 41 amino-acid peptide [3], [4].

Despite their critical roles in HPA axis function, adequate and robust tools for specifically visualizing and targeting the neurons that manufacture and release CRH have lagged behind those for other cell types. In part, the paucity of accumulated CRH peptide in cell bodies has necessitated the use of additional tools such as colchicine [5] for identifying cells, but this approach makes physiological experiments unreliable; in addition, experience dependent shifts in the expression of hypothalamic peptides [6], [7] hamper definitive identification of PNC phenotypes. While much of the field has employed the rat as a model for studying HPA physiology, recent advances have generated several transgenic approaches to assess CRH neurons through promoter-linked expression of eGFP or Cre recombinase [8], [9], [10], [11], [12]. None of these have specifically addressed or detailed the phenotype of labelled CRH neurons in the PVN. Given evidence for differences in HPA function and anatomical relationships of PVN cell populations [13] between mice and rats, a solid foundational understanding of the murine PVN is critical. Here we set out to investigate the adequacy of one such targeting strategy by examining the biochemical and electrophysiological properties of the PVN neurons it labels. We utilized a recently created and commercially available *Crh-IRES-Cre* knock-in line [8] which drives expression of Cre from the endogenous *Crh* promoter. We found that simple crosses of these founders with *Ai14* Cre-reporter mice [14] generated *Crh-IRES-Cre;Ai14* progeny with robust PVN expression of red tdTomato fluorescence. tdTomato expressing PVN neurons were easily and directly visible in live and fixed tissue. These cells were CRH-producing and stress responsive. tdTomato neurons exhibited intrinsic and synaptic electrophysiological properties consistent with those reported for rat PNCs, and could be targeted for cell-type specific expression of channelrhodopsin.

## Materials and Methods

All animal experiments were approved by the University of Calgary Animal Care & Use Committee (Protocol M09127), in accordance with the Canadian Council for Animal Care.

### Animals

B6(Cg)-Crh^tm1(cre)Zjh^/J (*Crh-IRES-Cre*)mice and B6.Cg-Gt(ROSA)26Sor^tm14(CAG-TdTomato)Hze^/J (*Ai14*) mice, whose generation has been detailed previously [8], [14], were obtained from Jackson laboratories (stock number 012704 and 007914 respectively). These were maintained as colonies of homozygous mice, with one backcrossing to C57BL/6J background strain following their arrival. Genotyping was used to identify mutants using PCR procedures provided by the supplier. The following primers were used to identify *Crh-IRES-Cre* mutants: 5′-CTT ACA CAT TTC GTC CTA GCC and 5′- CAA TGT ATC TTA TCA TGT CTG GAT CC-3′ and (468 base pair resultant PCR band). To identify *Ai14* mutants: 5′-GGC ATT AAA GCA GCG TAT CC-3′ and 5′-CTG TTC CTG TAC GGC ATG G -3′ were used (196 base pair band). Mice were housed on a 12∶12 hour light: dark schedule (lights on at 7:00) with ad libitum access to food and water. Pairs of either homozygous *Crh-IRES-Cre* or *Ai14* genotypes were mated, and the resulting heterozygous *Crh-IRES-Cre;Ai14* male offspring used in subsequent experiments. For stress experiments, mice were exposed to forced swim stress (between 8:00–9:30) consisting of 15 min in a glass cylinder (14 cm internal diameter) filled with 30–32°C water. Naïve littermates were used for comparison.

### Immunohistochemistry

8 week old *Crh-IRES-Cre^+/−^;Ai14^+/^* ^−^ mice were given a unilateral intracerebroventricular infusion of colchicine (80 µg in 4 µL saline) under ketamine/xylazine anesthesia. 18–22 hours later they were sacrificed. To label neuroendocrine cells, some mice were give a single intraperitoneal injection of fluorogold (2% w/v in 100 uL saline; Fluorochrome) and sacrificed 5 days later. To prepare fixed brain tissue, mice were anesthetized with sodium pentobarbital (30 mg/kg) and transcardially perfused with phosphate-buffered saline (PBS), followed by 4% paraformaldehyde (PFA) in phosphate buffer (PB, 4°C). Brains were placed in PFA 24 hours followed by 20% sucrose PB. 30 µM coronal brain sections were obtained via cryostat in 3 series. Rinses were performed before/between incubations with tris-buffered saline containing triton (TBSt; pH 7.4, with 0.1% Triton-X 100), Blocking solution (5% normal donkey serum in TBSt) was pre-applied for 1 hour and used in subsequent antibody incubations. Primary antibodies used were: rabbit anti- c-Fos Ab5 (1∶10,000 dilution; overnight RT; Calbiochem), rabbit anti-fluorogold (1∶10,000; Chemicon), monoclonal mouse anti- Oxytocin or Vasopressin (1∶10,000; PS38 and PS41 from Dr. H. Gainer; overnight RT;), rabbit anti-somatostatin (1∶1000; Cat. #20067 Immunostar) rabbit anti- preproTRH (1∶2000; 363J from Dr. M. Wessendorf) or rabbit anti-CRH (1∶4000; C-70 from Dr. W. Vale). Secondary antibodies/conjugates used were: Alexa-488-conjugated donkey anti-mouse (1∶500; Molecular Probes), Alexa-488 donkey anti-rabbit, biotinylated donkey anti-rabbit (1∶500; Jackson Immuno Research), and Alexa-488-conjugated streptavidin (1∶500; Molecular Probes). Slide-mounted and coverslipped sections were imaged using a confocal microscope (Olympus BX50 Fluoview). We included for assessment the entire rostral-caudal extent of one side of the PVN. Immunoreactive and tdTomato+ soma were counted using ImageJ.

### Optogenetics

In a stereotaxic apparatus under isoflurane anesthesia, glass capillaries were lowered into the brain of 6 week old Crh-IRES-Cre;Ai14 mice (anteroposterior, 0.0 mm; lateral, −0.3 mm from the bregma; dorsoventral, −4.5 mm from the dura). Recombinant AAV carrying ChR2-eYFP (Addgene plasmid 20298, pAAV-EF1a-double floxed-hChR2(H134R)-EYFP-WPRE-HGHpA) [15] was pressure unilaterally injected with Nanoject II apparatus (Drummond Scientific Company) in a total volume of 210 nl (3.4×10^13^ GC ml^−1^). Mice were allowed to recover >14 days before experiments. To excite channelrhodopsin in *in vitro* slices (preparation described below), a fiber optic cable (105 µm core diameter) was placed 1–2 mm from the PVN using a manipulator to deliver light from a laser (473 nm, OptoGeni 473, IkeCool Corporation). Light intensity was measured by a Photodiode Power Sensor (Thorlabs). Maximally, 2.5 mW light was delivered to tissue.

### Electrophysiology

Young male *Crh-IRES-Cre;Ai14* mice (4–6 weeks postnatal) were deeply anaesthetized with isoflurane and decapitated; brains were rapidly removed and immersed in ice cold slicing solution containing, in mM: 87 NaCl, 2.5 KCl, 0.5 CaCl_2_, 7 MgCl_2_, 25 NaHCO_3_, 25 D-glucose, 1.25 NaH_2_PO_4_, 75 sucrose saturated with 95% O_2_/5% CO_2_. 250 µm coronal sections were obtained using a vibratome (Leica), and allowed to recover for 1+ hours in 95% O_2_/5% CO_2_ saturated, 30°C artificial cerebrospinal fluid (aCSF) containing (in mM): 126 NaCl, 2.5 KCl, 26 NaHCO_3_, 2.5 CaCl_2_, 1.5 MgCl_2_, 1.25 NaH_2_PO_4_, 10 glucose. All recordings took place in aCSF at 30–32°C perfused at a rate of 1 mL/min, with DNQX (10 µM, Tocris) or picrotoxin (100 µM, Sigma) applied via perfusion pump. Neurons were visualized with an upright microscope fitted with differential interference contrast and epifluorescence optics (UVICO, RappOptoElectronics) and camera (AxioCam MRm). Borosilicate pipettes (3–5 mΩ) were filled with internal solution containing (in mM) 108 K-gluconate, 2 MgCl_2_, 8 Na-gluconate, 8 KCl, 1 K_2_-EGTA, 4 K_2_-ATP, 0.3 Na_3_-GTP, 10 mM HEPES, 0.2 Alexa-488 hydrazide and 10 mg·mL^−1^ biocytin. A monopolar aCSF-filled electrode placed about 20 µm from the cell was used to evoke pairs of post-synaptic currents (IPSCs) 50 milliseconds apart at 0.2 Hz intervals. Signals were amplified (Multiclamp 700B, Molecular Devices), low pass filtered at 1 kHz, digitized at 10 kHz (Digidata 1322, Molecular Devices), and recorded (pClamp 9.2, Molecular Devices) for offline analysis of evoked or spontaneous synaptic currents (Clampfit, Molecular Devices; MiniAnalysis, Synaptosoft). Post-recording slices, after fixation in 4% PFA (24 hours), incubation with streptavidin-A488 (1∶500 TBSt),and clearing using 50∶50 glycerol: TBS, were mounted and imaged via confocal.

### Data Analysis/Statistics

Where quantification was made, data are represented as mean ± standard error of the mean (s.e.m.). Statistical analysis was made in GraphPad Prism 4 using an unpaired student's t-test to for two group comparisons. P values less than 0.05 were considered significant.

## Results

**Figure 1:**
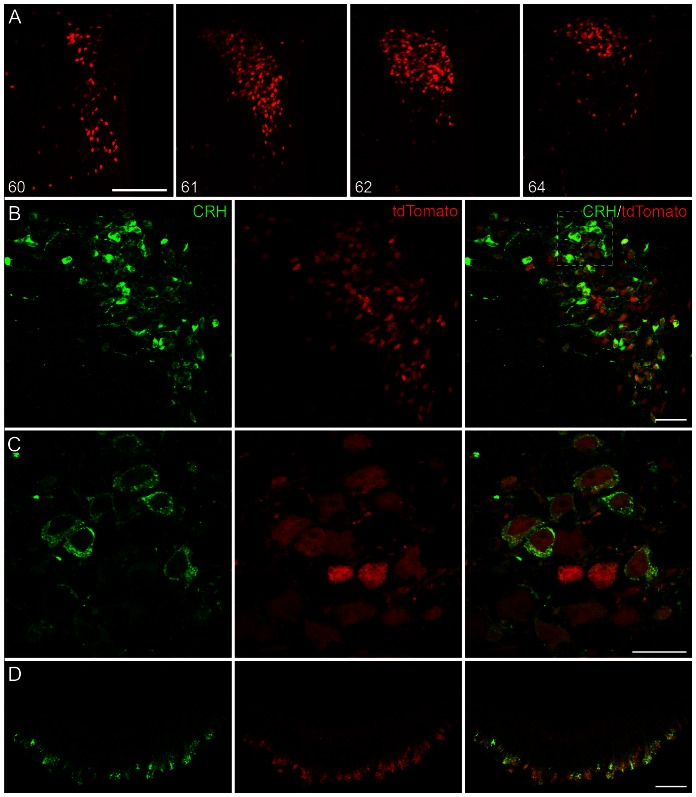
Anatomical distribution and CRH protein expression in tdTomato cells in the PVN of *Crh-IRES-Cre;Ai14* mice. **A**) Confocal images (20× magnification) of the rostral-caudal extent of the PVN in a naïve tdTomato mouse. In lower left corner, Allen Brain Atlas level is indicated. **B**) confocal image (40× magnification) of a colchicine-treated *Crh-IRES-Cre;Ai14* mouse PVN. In green, immunostaining against corticotropin-releasing hormone (CRH) is shown.**C**) Higher magnification of the (100×) image of the box inset in (B). **D**) Colocalization of CRH immunoreactivity and tdTomato at the external zone of the median eminence, shown at 40× magnification. Scale bars are 100 µm (A), 50 µm (B, D), and 20 µm (C).

We utilized heterozygous offspring derived from crossing homozygous *Crh-IRES-Cre* and *Ai14* mice. In fixed or live tissue, robust intracellular expression of tdTomato was observed in PVN as well as in other brain structures known to express CRH protein. These include the cortex, bed nucleus of the stria terminalis, and central nucleus of the amygdala. Here we focused exclusively on further characterizing tdTomato neurons in the PVN using immunohistochemistry, electrophysiology and optogenetics. tdTomato somata formed a compact cluster in the PVN (Fig. 1A). This expression was consistent and robust amongst all tested offspring using this mating scheme; all subsequent experiments were performed without requiring any secondary detection methods for amplifying tdTomato fluorescence.

### PVN neuropeptide expression profile of *Crh-IRES-Cre;Ai14* tdTomato neurons

**Figure 2:**
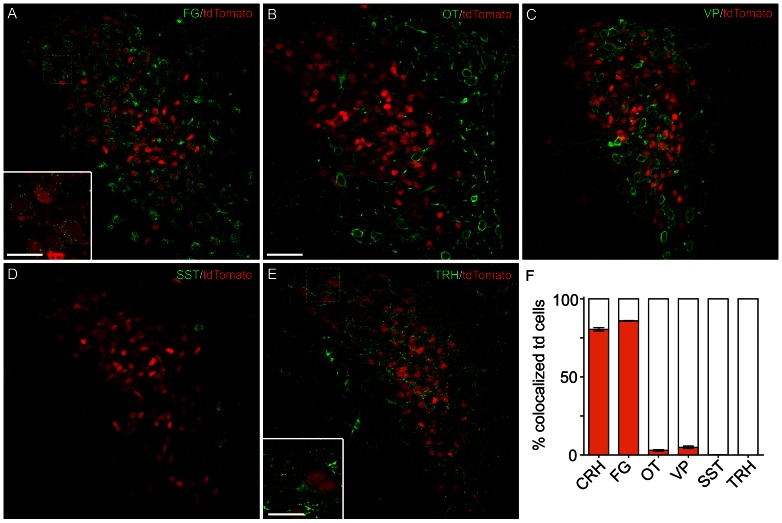
Neurosecretory and neuropeptide phenotype of tdTomato cells in *Crh-IRES-Cre;Ai14* mice. **A**) Retrograde transport of peripherally-delivered fluorogold in tdTomato neurons. A confocal image (40× magnification) shows tdTomato in the PVN (red) along with fluorogold immunoreactivity. Higher magnification (100×) image indicated by box is shown in lower left corner. Neuropeptide immunoreactivity for **B**) oxytocin (OT), **C**) vasopressin (VP), **D**) somatostatin (SST), or **E**) thyrotropin-releasing hormone (TRH; with 100× magnification inset) in *Crh-IRES-Cre;Ai14* mouse PVN, shown at 40× **F**) Bar graph showing the percent of tdTomato positive cells that coexpress each neuropeptide, each from n = 5 colchicine-treated mice. Graphed data are represented by mean ± SEM. Scale bars are 50 µm (A-E large), 20 µm (A, E inset).

Using a Cre-based strategy to define cell groups by genetic identity, while robust, has the potential to “mis-label” neuronal populations [16]. This can occur either because of “leaky” transgenic constructs, or because transient gene expression during development is indistinguishable from sustained gene expression in adults using this strategy [17], [18]. Data available online from the Allen Brain Atlas [18] show that mRNA expression of Cre-recombinase or tdTomato in the *Crh-IRES-Cre;Ai14* mice, at various ages, is relatively consistent with that of *Crh* in many brain regions, suggesting that this *Crh-IRES-Cre;Ai14* strategy identifies cells with endogenous promoter-driven *Crh* gene expression. To further test the adequacy of this method specifically for labeling CRH neurons in the PVN, we examined the overlap of tdTomato expression with CRH protein in adult mice. We performed immunohistochemistry in hypothalamic slices from *Crh-IRES-Cre^+/−^; Ai14^+/−^* mice (n = 5) given i.c.v. colchicine to enhance somatic accumulation of neuropeptides. CRH immunoreactivity was observed in 80.5±1.1% of the tdTomato neurons (Fig. 1B, C, 2F). Conversely, nearly all somata containing CRH co-expressed tdTomato (96.0±0.3%). In addition to this, we found that tdTomato was present at the external lamina of the median eminence, where it also colocalized with CRH protein (Fig. 1D). Given the high degree of colocalization of CRH with tdTomato both in the PVN and at the median eminence, we next asked what percentage of tdTomato neurons would accumulate peripherally-delivered retrograde retrogradely tracer, fluorogold (FG). If these cells were indeed neuroendocrine neurons, their axon terminations would be outside the blood-brain barrier and should readily take up FG. In mice given an intraperitoneal injection of FG 5 days prior to sacrifice, 85.9±0.2% of tdTomato neurons exhibited FG immunoreactivity (n = 3 mice; Fig. 2A, F). Together these results indicate that adult PVN neurons in *Crh-IRES-Cre;Ai14* mice largely represent a population of CRH-producing cells in the PVN, and the overwhelming majority of these are neuroendocrine cells.

In addition to CRH, the PVN contains many distinct neurotransmitter-expressing populations that serve discrete functions. Magnocellular neuroendocrine cells (MNCs), which are perhaps the best characterized, produce the neuropeptide hormones oxytocin (OT) and arginine-vasopressin (AVP), which are secreted from terminals in the posterior pituitary into peripheral blood. More critically, PVN neuroendocrine cells share a developmental origin, of which CRH-containing PNCs and MNCs are most similar [19], and colocalization of OT or AVP with CRH has been described in rats under various physiological conditions [7], [20], [21]. In sections from colchicine-treated mice (same mice as above; n = 5) probed with an antibody against the OT, we found scarce colocalization (3.0±0.5% of tdTomato+ cells; Fig. 2B, F). Most OT+ cells were generally located medially or ventrally to tdTomato neurons, with little spatial overlap. Interestingly, of all dual OT+/tdTomato+ cells, most were located in the caudal aspect of PVN (Allen reference atlas level 64–66 [13], [18]; 69.4±6.5% of all OT+/tdTomato+ cells) of the PVN. In more rostral sections, the remaining OT+/tdTomato+ neurons were usually located ventrally. Cells exhibiting AVP immunoreactivity were generally distributed, amongst tdTomato+ cells, with slight medial preference in rostral PVN. Despite this physical proximity, we found little colocalization of tdTomato with AVP (4.9±0.8% of tdTomato+ cells; Fig. 2C, F). We also examined two other major neuroendocrine cell populations that have been described within the PVN: somatostatin (SST) and thyrotropin-releasing hormone (TRH) [22]. We found virtually no overlap of tdTomato expression with SST (3 colocalized cells in 5 mice; Fig. 2D,F) or TRH (0 colocalized cells; 5 mice; Fig. 2E,F) immunoreactive cells. While non-somatic TRH immunoreactivity in putative dendrites/axons was observed in close proximity to tdTomato neurons, higher magnification images confirm that these structures are distinct (Fig. 2E). Together these data suggest that *Crh-IRES-Cre* neurons exhibit strong overlap with CRH, but very limited/no overlap with other classical PVN neuropeptide markers.

### Activation of *Crh-IRES-Cre;Ai14* tdTomato neurons following *in vivo* stress

**Figure 3:**
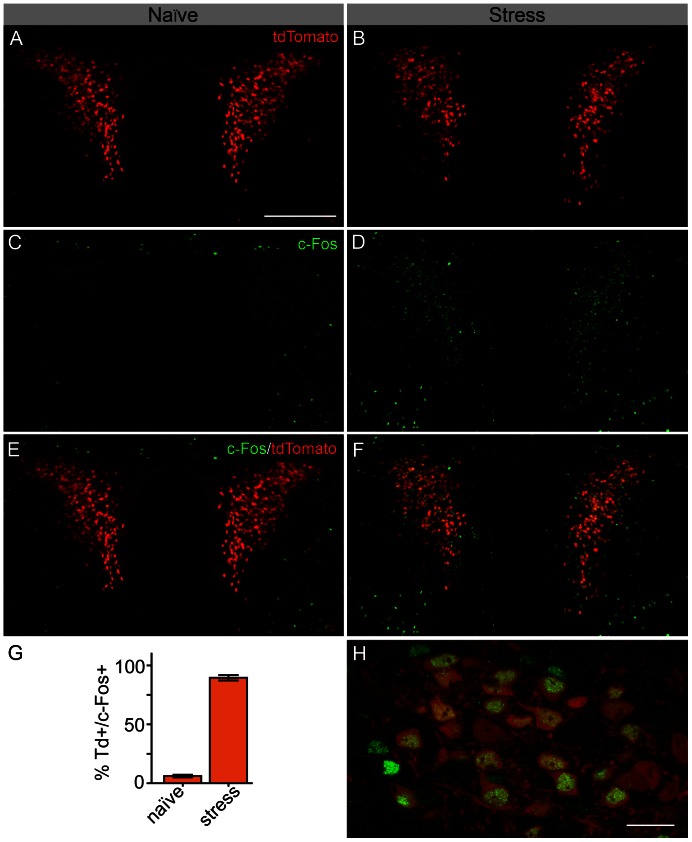
Induction of c-Fos protein in PVN tdTomato neurons following stress. **A,B**) tdTomato expression in the PVN of naïve *Crh-IRES-Cre;Ai14* mice, and one exposed to a 15 min forced swim stress, 90 min prior to sacrifice. Confocal image at 15×. **C,D**) c-Fos immunoreactivity in these naïve or stress brain sections, showing an increased number of c-Fos containing nuclei following stress. **E,F**) Merged image showing high spatial colocalization of c-Fos and tdTomato in the stressed, but not naïve, mouse. **G**) Bar graph summarizing changes in the percentage of tdTomato neurons co-expressing c-Fos in naïve (n = 5) versus stressed (n = 6) mice. **H**) Higher magnification (100×), of c-Fos immunoreactivity in tdTomato cells from a stressed mouse. Data summarized in (H) are mean ± SEM. Scale bars are 200 µm (A–F) and 20 µm (H).

In response to a variety of stress challenges, PNCs are subjected to increases in synaptic drive and exhibit an increase in neuronal activity, as action potential propagation into axon terminals at the median eminence is a necessary step for secretion of vesicle-bound pituitary releasing hormones like CRH. A history of robust neuronal activity can be observed through increases in PVN cellular expression of the immediate early gene c-Fos [23], [24]. We asked to what extent tdTomato-expressing neurons in the PVN would respond to stress with increases in c-Fos expression. 90 min following a 15 min forced swim stress, mice and naïve littermate controls were sacrificed. We performed c-Fos immunohistochemistry and quantified double positive c-Fos and tdTomato neurons (Fig. 3). We observed robust induction of c-Fos expression in the PVN. More importantly we noted that, following stress, 89.5±2.3% of tdTomato+ neurons contained c-Fos (n = 6; Fig. 2B, D, F–H). This was significantly different from tdTomato neurons from naïve mice, where c-Fos expression and cFos/tdTomato coexpression was low (6.1±1.1%; n = 5; p<0.0001 vs. stress, unpaired t-test; Fig. 3A, C, E, G).

### Morphological and electrophysiological profile of *Crh-IRES-Cre;Ai14* tdTomato neurons

**Figure 4:**
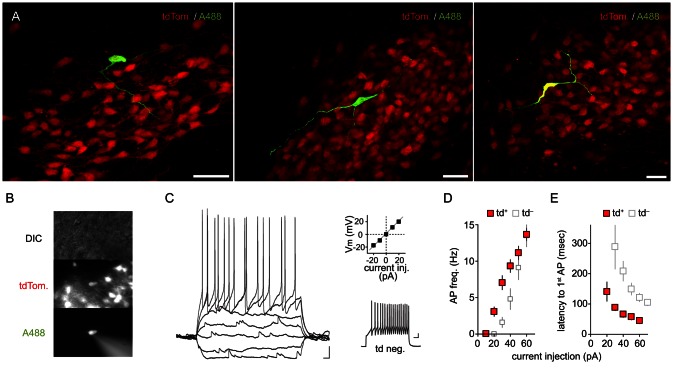
Morphological and intrinsic membrane properties of *Crh-IRES-Cre;Ai14* tdTomato neurons. **A**) Confocal Images (60× magnification), from 3 mouse PVN sections, showing morphology of single tdTomato neurons filled with Alexa488- and biocytin during whole-cell patch clamp recordings. **B**) Epifluorescence and differential interference contrast (DIC) images during whole-cell recording from a tdTomato positive PVN neuron. **C**) Left: current clamp trace of a tdTomato positive neuron. Membrane potential was −80 mV, current injection started at −20 pA with 10 pA increments. Scale bar: 0 mV/100 msec. Right,above: current-voltage plot for current injection (0±20 pA, Δ10 pA) in tdTomato neurons (n = 23 cells). Right,below: 50 pA current injection from −80 mV in a tdTomato negative neuron. Scale: 0 mV/100 msec **D**) Graph of action potential frequency for varied positive current steps from −80 mV in tdTomato+ (n = 23) and large soma tdTomato- (n = 15) neurons. **E**) Latency (in msec) to first action potential for varied current injection steps in tdTomato+/− neurons.

In rats, PVN neuronal cell types have been characterized using electrophysiological methods [25], [26], [27]. Despite this, certainty about the transmitter phenotype has not been as robust or successful for non-OT/AVP cell types. Some studies, in rats, have confirmed some PNC- or CRH neuron- specific biophysical properties by relying on laborious single cell RT-PCR, or *post-hoc* immunohistochemistry [28], [29], [30], [31], but a detailed description of a murine equivalent has been lacking. Thus, we investigated whether *Crh-IRES-Cre^+/−^;Ai14^+/−^*mice would delineate an electrophysiologically homogenous cell population. We obtained whole-cell recordings from tdTomato+ neurons in acute *in vitro* hypothalamic slices. Alexa-488 hydrazide and biocytin were included in the patch pipette as morphological indicators for confirming cell tdTomato expression and morphology in real time and for *post hoc* analysis (Fig. 4A). We observed that tdTomato neurons (n = 8) were morphologically simple, consistent with descriptions for rat PNCs [32], [33], [34]. From their fusiform somata we noted the emergence of two or three processes. In a few cases, putative axons could be observed to penetrate deep into the slice, hundreds of microns away from the PVN. Some putative dendrites exhibited additional bifurcations, and some possible spine-like structures.

tdTomato+ neurons (Fig. 4B) display many electrophysiological characteristics similar to rat PNCs, including a relatively high input resistance (932.9±19.8 MΩ, n = 23) and near linear input-output current-action potential relationship with moderate spike-frequency adaptation (Fig. 4C–D). Latency to a first action potential was short, decreasing further with incremental depolarizing current steps from −80 mV. With a 60 pA depolarizing current step, latency was 45.4±6.8 msec (n = 23; Fig. 4E). Time to onset of firing in response to a depolarizing step was shorter than that observed in larger diameter tdTomato-negative cells (Fig. 4C lower right) which consistently expressed a stereotyped delay to first spike (121.4±13.8 msec at 60 pA; P<0.0001 vs. tdTomato+; Fig. 4E). This phenotypic difference in action-potential latency is consistent with low expression, in PNCs, of the transient inward voltage-gated potassium current that delineates MNCs in rats [27]. We failed to observe, at least in the mid-rostral/caudal-level PVN where most of our recordings were obtained, low-threshold spikes which can be observed in rat neurons projecting to other central structures [26].

**Figure 5:**
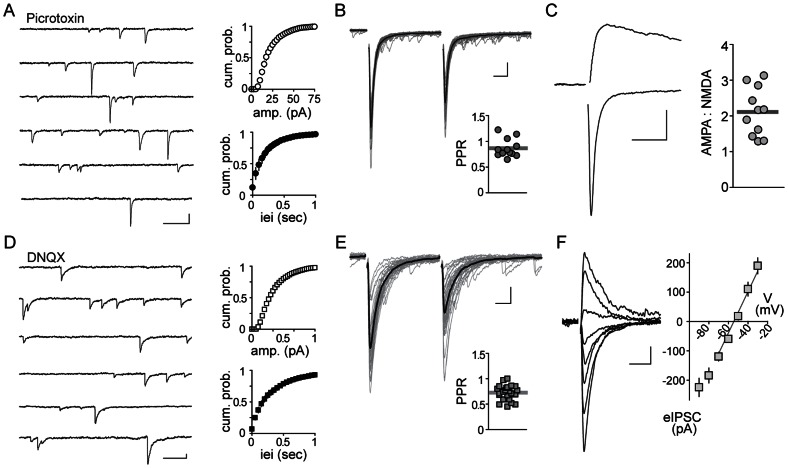
Fast glutamate and GABA transmission in Crh-IRES-Cre;Ai14 tdTomato neurons. **A**) Left: Sample recording, in voltage-clamp mode, from a single tdTomato neuron in the presence of picrotoxin (100 µM) blocking GABAA receptors. Right: plots, obtained from analysis of 5 min recording showing a cumulative distribution of amplitudes (top) and inter event intervals (iei's) of spontaneous EPSCs during this period. **B**) Above: Averaged (black) and un-averaged (grey) evoked EPSCs. Paired-pulse interval is 50 msec. Below: Data from n = 12 tdTomato neurons showing paired-pulse ratio (PPR: evoke 2/evoke1). **C**) Left: Sample eEPSC traces recorded from an individual td+ cell at −80 mV (lower inward AMPAR-mediated current) and at +40 mV after addition of DNQX (10 µM; upper outward NMDAR current). Right: Ratio between inward AMPAR and outward NMDAR currents for n = 11 tdTomato neurons in the PVN. **D**) Left: spontaneous GABAAR-mediated inhibitory post-synaptic currents (IPSCs) recorded at −80 mV in a single td+ neuron with 10 µM DNQX. Right: cumulative distribution plots from this cell, of IPSC amplitudes and iei's. **E**) Above: evoked IPSCs with 50 msec interval. Averaged (black) and individual trials (grey) overlaid. Bottom: PPR data from n = 17 tdTomato neurons. **F**) Left: individual traces of evoked IPSCs (eIPSCs), in a td+ cell, recorded at varied holding potentials (−100 mV to −30 mV, 10 mV increment). Right: eIPSC current-voltage relationship showing eIPSC reversal potential in n = 6 td+ neurons. Data are represented by mean ± SEM. Scale bars 20 pA/50 msec in (**A,D**) and 50 pA/10 msec in (**B,C,E,F**).

We next characterized fast glutamatergic and GABAergic transmission onto tdTomato+ neurons. We obtained whole-cell, voltage-clamp recordings in the presence of picrotoxin, and observed spontaneous excitatory post-synaptic currents (EPSCs) (amplitude: 23.5±1.6 pA; frequency: 7.3±2.6 Hz; n = 12; Fig. 5A). We could also evoke larger, action potential-dependent EPSCs (eEPSCs) by electrical stimulation of the surrounding neuropil (Fig. 5B). Similar to observations in rat PNCs [35], these excitatory synapses mostly exhibited paired-pulse depression when two stimuli were delivered with a 50 msec interval (paired-pulse ratio/PPR: 0.86±0.05; n = 12; Fig. 5B). These events were completely absent in the presence of the AMPA/KA receptor antagonist, DNQX (10 µM). In addition to AMPAR mediated EPSCs, these synaptic inputs also exhibited an NMDA receptor (NMDAR)-mediated current component that was unmasked at depolarized holding potentials in the presence of DNQX (AMPA∶NMDA ratio: 2.1±0.2; n = 11; Fig. 5C). To examine fast GABA transmission, we conducted experiments in the absence of picrotoxin, but with DNQX in the bath. At a holding potential of −80 mV, and with 12 mM chloride in the standard intracellular solution, we observed spontaneous GABAA-receptor (GABAAR)-mediated inward currents (amplitude: 27.6±2.1 pA; frequency: 4.0±0.63 Hz; n = 17; Fig. 5D) and could evoke action potential driven events that showed consistent paired-pulse depression (paired-pulse ratio: 0.73±0.03; n = 17; Fig. 5E). These currents reversed at −54.3±0.8 mV (Fig. 5F), consistent with GABA IPSCs in naïve PNCs in rats using 12 mM intracellular Cl^−^ [36]. Together, these electrophysiological findings provide the first definitive characterization of the cellular and synaptic properties of CRH neurons in the PVN. Robust expression of tdTomato in live PVN tissue of *Crh-IRES-Cre^+/−^;Ai14^+/−^* provides an improved and reliable method of cell identification for future patch-clamp studies of synapses and excitability of CRH neurons.

### Optogenetic manipulation *of Crh-IRES-Cre* neurons

**Figure 6:**
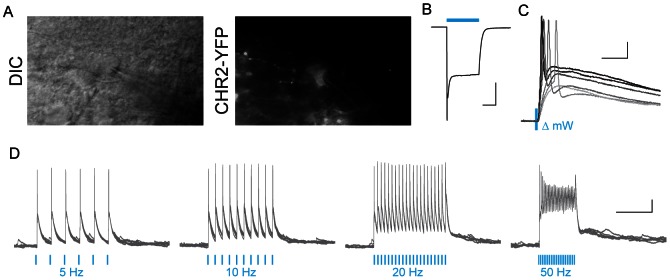
Optogenetic activation of Crh-IRES-Cre PVN neurons by cre-dependent viral expression of channelrhodopsin. **A**) DIC and epifluorescence images showing whole-cell recording from a YFP+ PVN neuron. **B**) example of a photocurrent recorded in voltage-clamp mode, elicited by 473 nm blue light exposure in the same YFP+ neuron. Light was delivered through a fibre optic cable end placed near the PVN **C**) examples of light-induced action potentials in a PVN neuron, and the variation of action potential waveform with varied light intensity. **D**) Trains of light pulses (2 msec pulse-width) at various frequencies and resulting action potentials. Scale bars are 500 pA/100 msec in (B), 10 mV/10 msec in (C), and 10 mV/500 msec in (D).

Finally, we investigated the feasibility of utilizing *Crh-IRES-Cre* mice as a tool to optogenetically target CRH neurons. Using local PVN microinjection of an AAV containing a floxed channelrhodpsin (ChR2) construct which is expressed in a Cre-dependent manner, we were able to infect PVN neurons and visualize YFP-tagged ChR2 in these cells in hypothalamic slices (n = 8; Fig. 6A). YFP+ neurons exhibited intrinsic membrane properties in whole-cell recordings that are indifferent from those reported above for tdTomato neurons (data not shown). Using a fibre optic to deliver 473 nm blue light to the slice, we elicited large photo-currents (range: 200 pA to 1.5 nA; Fig. 6B). Short pulses (1–5 msec), with varying light power (0.5 mW increments) could efficiently drive single action potentials (Fig. 6C). Trains of 473 nm light pulses reliably elicited action potentials at frequencies up to 20 Hz (Fig. 6D). Plateau potentials occurred at frequencies 50 Hz and higher. These data indicate that specific optogenetic manipulation of CRH neurons is an effective and potentially useful strategy for studying the physiological consequences of CRH neuronal activity.

## Discussion

Our study of *Crh-IRES-Cre^+/−^;Ai14^+/−^* mice validates a simple strategy to directly visualize and optogenetically control stress-responsive and CRH-producing neurons in the PVN. These mice exhibit robust cellular expression of tdTomato fluorescence highly colocalized to CRH protein in the PVN and the median eminence. Moreover, tdTomato expression was distinct from the expression of OT, AVP, TRH or SST. Following a forced swim stress, the vast majority of tdTomato neurons express the cell activation marker c-fos, suggesting that tdTomato reporting reliably targets a highly stress-responsive neuronal population. Finally, *Crh-IRES-Cre^+/−^;Ai14^+/−^* tdTomato expressing cells in the PVN are highly amenable to both electrophysiological and optogenetic study. Together, these data offer, to the best of our knowledge, a first detailed description of combined morphological, biochemical, and electrophysiological properties of identified CRH neurons in the mouse PVN.

The emergence of numerous Cre-loxP-based approaches directed towards reliably identifying CRH neurons highlights the rapid development of tools in this research area [9], [10], [11]. While each of these transgenic/mutant mouse lines holds value, a thorough characterization of the target population of PVN neurons has been missing. The use, here, and in one other study [12], of recently described knock-in CRH Cre-driver line in combination with *Ai14* reporter mice may also hold advantages over some previous strategies. First, because Cre is inserted immediately following the endogenous *Crh* gene locus, Cre expression may occur in a manner more similar to native promoter-driven *Crh* expression [8]. Second, use of the *Ai14* Cre-reporter line results in unparalleled morphological detail of labeled neurons, and is one of the most robust reporters currently available [14]. Indeed, we consistently observed abundant tdTomato levels in animals heterozygous for both *Crh-IRES-Cre* and *Ai14* alleles in both live and fixed tissue, with no need for signal amplification.

Importantly, tdTomato expression overlaps extensively with CRH immunoreactivity in both PVN and median eminence. Expression of tdTomato was nearly universal in CRH immunopositive soma, suggesting extremely efficient *Crh* promoter-driven Cre recombination. Conversely, while expression of CRH in PVN tdTomato neurons was very high, tdTomato cells were more abundant. This is not surprising, as immuno-identification of CRH neurons is thought to under-represent the total CRH neuronal population in PVN. Even with a very high dose of colchicine, it is reasonable to expect that Cre-loxP labeling sensitivity may exceed that of antibody-based CRH protein detection in reliably identifying CRH-positive somata. Furthermore, Cre-mediated recombination, and thus tdTomato expression occurs cumulatively over development – effectively reflecting a cellular history of *Crh* gene transcription; by contrast, immunohistochemical identification of CRH neurons provides a snapshot in time of the current protein content above a detection threshold. Exploiting the fact that tdTomato expression tracks cellular expression of CRH over the lifespan off the animal could be interesting in the context of perinatal exposures to stress [31], [37]. Importantly, because we observed extensive c-Fos expression in these neurons, this method should prove useful for identifying a functionally homologous population of stress-related neurons independent of CRH synthesis levels. These data, together with *in situ* hybridization studies publicly available for this line [18] support the idea that *Crh-IRES-Cre;Ai14* mice are a valid tool for marking *bonafide* CRH-producing neurons. As noted above, colchicine treatment has been necessary for identifying CRH neurons and examining their morphology in the past [34]. As colchicine is toxic at typically used doses, and thus is a profound physical stress [23], it is plausible that CRH neurons examined using this approach may have undergone adaptive morphological changes. Thus, this mouse model could be extremely useful in future anatomical studies of CRH neurons both by reducing animal suffering and the confounding effects of colchicine treatment on stress physiology.

The structure and organization of the rat PVN has been repeatedly described, and meticulously detailed in recent years [38]. In contrast, few descriptions exist for mice. In agreement with one such study by Biag et al [13], we, too, noted that the spatial relationship of parvocellular (CRH/tdTomato) and magnocellular (OT/VP) regions of the PVN appears to be inverted compared to the rat, with tdTomato neurons found more laterally in the mouse PVN. Taken together with these previous descriptions, our observations point to a fundamental architectural difference in PVN between these two commonly used rodent species. On the other hand, some similarities also exist. For example, co-expression of tdTomato with either OT or VP was very low in these animals, which coincides with reports from adrenally intact or non-stressed rats [7], [38]. In rats, CRH colocalization with VP in PNCs has only been reported either when glucocorticoids are removed [39] or after repeated stress [40], [41]. CRH colocalizes with OT in magnocellular neurons during cellular dehydration [42]. These types of colocalization profiles have yet to be examined in mice. Anatomy notwithstanding, we found that many basal electrophysiological and synaptic properties of PVN cell types appear to be conserved between the two rodent models. tdTomato+ neurons exhibited short latency to action potential firing; in contrast, tdTomato^−^ neurons displayed a delay to onset of first spike in response to a depolarizing current step. This concurs with electrophysiological profiling of PNCs compared to MNCs in rat [25], [26], [27]. In addition, we observe high release probability at both glutamatergic and GABAergic inputs onto tdTomato neurons. This is also consistent with that seen in rat PNCs and further confirms that synapses on CRH neurons may effectively function as low-pass filters under naïve conditions [43]. These data suggest, overall, a conserved method of information transfer and processing for CRH neurons across different species.

The extent to which the homology of intrinsic and synaptic properties in naïve rodent PVN neurons extends to stress-relevant synaptic [44], cellular, or system level physiology remains to be seen. We hypothesize that an additional advantage of *Crh-IRES-Cre;Ai14* mice is the ability to reliably identify a stress-relevant neuronal population following acute or chronic stress experiences. Thus far, we have been obliged to assume that the intrinsic properties used to identify PNCs are static. Given that neuropeptide expression, for example, undergoes dramatic changes with physiological manipulation [39], [45], this assumption may not be accurate. In terms of stress-associated synaptic changes, the current literature suggests that at least one change is conserved: In both rats and mice, stress exposure causes alterations in PNC chloride homeostasis, resulting in excitatory GABA transmission [10], [36]. While the basic function of the HPA is preserved in diverse organisms: from teleost fish to human beings, several modifications have been made through evolution [46]. Indeed, HPA adaptations to repeated/chronic stress challenges, usually described in conventional rat models, are only sometimes studied or replicated in mice [47], [48], [49], [50]. Resulting discrepancies are difficult to interpret in relation to the value of specific animal models in their relevance to stress resilience or susceptibility to disease in humans, they highlight a need for multiple model organisms, including mice, in future research.

In addition to characterizing CRH-producing neurons in PVN, we show successful virus-driven expression of an exogenous protein: channelrhodopsin to highlight the utility of *Crh-IRES-Cre* mice for study and targeting of CRH neurons through the Cre-loxP method. While channelrhodopsin in particular has garnered recent attention in studies of stress/fear circuits [51], the ultimate purpose of this experiment is to exemplify one targeted manipulation of many that have recently been created to expand the Cre-loxP “toolbox” for neuroscientists. One can envision, for example, that a similar viral or transgenic strategy using DREADDs [52] or halo-/archeo-rhodopsins [53] to, instead, silence CRH neurons during a stress may reveal specific contributions and/or dynamic interplay of HPA or higher stress circuits in stress adaptation. Alternatively, cell specific knock-out of single genes and gene products [54], or genetically-encoded tracers of ionic/voltage indicators [55], [56] could be achieved through many of the “floxed” genetic constructs becoming increasingly available. Thus, utilization of tools such as the *Crh-IRES-Cre* mouse will undoubtedly increase in their value as the field moves towards tackling causal questions in brain and body function, and particularly in our understanding of neuroendocrine physiology.
